# Comprehension and Hemispheric Processing of Irony in Schizophrenia

**DOI:** 10.3389/fpsyg.2017.00943

**Published:** 2017-06-13

**Authors:** Ronit Saban-Bezalel, Nira Mashal

**Affiliations:** ^1^The School of Education, Bar Ilan UniversityRamat Gan, Israel; ^2^Gonda Multidisciplinary Brain Research Center, Bar Ilan UniversityRamat Gan, Israel

**Keywords:** schizophrenia, irony, divided visual field paradigm, hemispheres

## Abstract

Studies focusing on the comprehension of figurative language among schizophrenia patients (SZ) reveal their difficulties comprehending such language and their tendency to interpret it literally. The present study investigated hemispheric processing and comprehension of irony in 16 SZ patients and 18 typically developing (TD) adults. Two experimental tasks were used: an online divided visual field experiment and an offline irony questionnaire. The results show an atypical reversal of hemispheric processing of irony in SZ patients as compared to TD adults. While the TD group demonstrated a right hemisphere advantage in processing irony, SZ patients demonstrated a left hemisphere advantage. Greater comprehension of irony was associated with decreased negative symptoms. In addition, under conditions that not involving a time restriction, the SZ patients’ performance improved. Our findings reinforce those of previous studies suggesting that brain lateralization is atypical in SZ patients.

## Introduction

Figurative language serves many of our communicative goals, such as being eloquent or humorous, or clarifying our intentions and it thus common in everyday discourse (Roberts and Kreuz, 1994). Comprehending figurative language requires the listener to grasp the speaker’s communicative intention and go beyond the literal meaning of the words in the utterance (Berman and Ravid, 2010). The pragmatic ability to process non-literal language is an essential social skill that contributes to one’s well-being (Champagne-Lavau and Stip, 2010). Recently it has been shown that discourse and non-literal understanding is compromised in schizophrenia, with pragmatic deficit co-occurred with cognitive or socio-cognitive deficits in about 30% of the participants. Furthermore, quality of life was predicted by symptoms and by pragmatic abilities (Bambini et al., 2016).

Irony is one form of figurative language, which is important in creating social bonds (Clark and Gerrig, 1984) and serves as a linguistic means for expressing humor (Roberts and Kreuz, 1994) as well as criticism (Dews and Winner, 1995). Studies have shown that irony is common in everyday discourse (Gibbs, 2000) and frequent in computer-mediated communication (Whalen et al., 2013). Often ironic remarks convey the opposite message of their literal meaning (Gibbs et al., 2014), so that comprehending them depends on the hearer’s ability to recognize the speaker’s intentions (Winner et al., 1988). According to Grice (Grice, 1991) irony is based on meaning contradiction or negation. The speaker overtly violates the first Quality maxim in order to convey a meaning contradictory to the one the utterance seems to convey. In the current study we investigated this type of irony comprehension.

Studies that focused on comprehension of figurative language among SZ patients (Iakimova et al., 2010; for review see Thoma and Daum, 2006) reveal the patients’ difficulty in comprehending such language and their tendency to interpret it literally. Ziv et al. (2011) studied the social cognition of irony in SZ patients compared to typically developing (TD) controls. The results showed that individuals with schizophrenia demonstrated some ability to comprehend irony in simple conversations that do not demand advanced cognitive or affective abilities. The researchers noted that their conclusion must be treated with caution, since an experimenter read all the tasks to the participants and may have provided them with prosodic cues. A recent fMRI study examined auditory irony processing in SZ patients during remission from a neurolinguistics perspective (Schnell et al., 2016). Participants performed an irony task and an irony task with linguistic hint (i.e., a reference of the speaker’s mental state). Participants were asked to judge the scenarios as quickly as they could. Results indicated poorer comprehension of irony in SZ as compared to TD participants. Furthermore, whereas TD participants activated brain regions associated with non-literal language and theory of mind (ToM), SZ patients activated brain areas associated with auditory and linguistic processing. However, linguistic cues enhanced patients’ irony comprehension and a similar pattern of brain activation was observed in both groups. The current study will also assess irony comprehension using two different tasks that vary in their demands. Utilizing the Assessment Battery for Communication (ABaCo) (Bosco et al., 2012a), broad pragmatic abilities (e.g., direct and indirect speech acts, irony, deceit, violations of Grice’s maxims, topic management, and turn-taking) were examined on five scales (i.e., linguistic, extralinguistic, paralinguistic, context, and conversational) in patients with schizophrenia (Colle et al., 2013). The results show that SZ patient’s impairment extends to all the domains of pragmatic ability as compared to healthy controls, with irony being the most difficult task.

Difficulties in figurative language comprehension in SZ have been attributed to a variety of factors, including ToM and executive functions. Champagne-Lavau and Stip (2010) examined whether pragmatic difficulties, including the comprehension of metaphors and indirect requests, coexist with difficulties in metalizing skills or difficulties in executive functions. Participants with SZ showed specific difficulties in understanding non-idiomatic and idiomatic metaphors along with difficulties in understanding the other’s mind and difficulties with flexibility. However, while flexibility did not seem to play a role in performances of SZ participants for pragmatic, indirect requests and idiomatic metaphors seemed to depend on ToM ability. Varga et al. (2014) also found impaired ToM abilities in SZ patients. In that study metaphor and irony comprehension was tested among participants with SZ with good general neurocognitive skills (IQ above 106), and a subgroup of SZ participants with lower IQ (IQ below 106). The results showed that although SZ participants with lower-IQ were able to understand conventional metaphors they were impaired in unconventional metaphor and irony comprehension. In contrast, the higher-IQ SZ subgroup was able to comprehend both conventional and unconventional metaphors, and irony, probably using IQ-dependent compensatory mechanisms. Thus, it seems that SZ patients can compensate their impaired ToM skills at least to some extent with good neurocognitive functions and intact semantic processing in order to understand irony.

Hemispheric differences in figurative language comprehension have been widely reported. A theoretical framework that explains the unique contribution of each hemisphere to language, processing, including figurative language is termed the Fine vs. Coarse Semantic Coding Theory – FCSCT (Jung-Beeman, 2005). This theory states that the left hemisphere (LH) engages in fine semantic coding. Dominant semantic features of stimuli are given priority over uncommon interpretations, and a single interpretation of a word and several of its close associates are activated in the LH. In contrast, the right hemisphere (RH) engages in coarse semantic coding. Hence, distant semantic relations of words or utterances, including various interpretations of ambiguous words and non-literal interpretations of phrases, are activated in the RH (Jung-Beeman and Bowden, 2000; Jung-Beeman et al., 2000). The Graded Salience Hypothesis (GSH) (Giora, 1999, 2002, 2003) is complementary to the FCSCT. The GSH posits that salient meanings, which are determined mainly by the frequency of exposure and familiarity with the meaning, are given high priority during processing, regardless of either literality or contextual fit (Giora, 1999, 2002; Giora and Fein, 1999; Giora et al., 2007). The GSH argues that the degree of semantic salience (e.g., familiarity, frequency, prominence, conventionality, prototypicality, contextual independence) rather than the literal/figurative meaning of an utterance determines processing. That is, both figurative (e.g., highly familiar idioms) and literal meanings can be salient if they are stored in our mental lexicon. This model has some implications for the roles of the LH and RH in processing literal versus figurative expressions. It claims that the literal meanings of passages represent their salient meaning. In contrast, ironic interpretations are based on decoding contextual cues in addition to literal meanings and thus are considered non-salient and should be activated faster in the RH.

These theoretical models have been supported by behavioral and neuroimaging studies that found RH specialization in processing non-salient interpretations of figurative language (Schmidt et al., 2007; Kasparian, 2013), including idioms (Mashal et al., 2008), novel metaphors (Faust and Mashal, 2007; Mashal and Faust, 2009) and irony (Eviatar and Just, 2006; Shibata et al., 2010; Saban-Bezalel and Mashal, 2015a) in TD adults. Hemispheric processing of irony was investigated with the divided visual field (DVF) paradigm in adults with autism spectrum disorders (ASD) compared to TD adults (Saban-Bezalel and Mashal, 2015a). The participants read short passages in which the final (target) word was missing. The target word provided either a literal or an ironic interpretation of the passage and was flashed randomly to the right visual field/LH or the left visual field/RH. The participants were asked to indicate whether the passage with the final word missing and the target word create a meaningful passage. In line with both the FCSCT and the GSH, the results showed faster responses in the RH as compared to the LH for the non-salient ironic stimuli in TD adults. In contrast, bilateral activation was observed in the ASD group when they processed ironic stimuli. In the current study we utilized the same paradigm to investigate comprehension and hemispheric processing of irony in SZ patients.

Atypical lateralization for language processing has been documented for individuals with SZ (Kircher et al., 2002; Mitchell and Crow, 2005). Rapp and Steinhäuser (2013) conducted a systematic review of fMRI studies on sentence- and text-level language comprehension in schizophrenia patients (SZ). The findings suggest functionally altered pathways for language processing in schizophrenia, that is, under-activation of a pre-dominantly left-lateralized frontotemporal network, as well as increased dual hemispheric activation. Indeed, studies that investigated novel metaphor processing (representing the non-salient interpretation) (Kircher et al., 2007; Mashal et al., 2013) have revealed a reversed lateralization in SZ. For instance, the comprehension of novel and conventional metaphors was investigated by Mashal et al. (2013). The results revealed reduced comprehension of both types of metaphors in the SZ group. Whereas TD adults activated the right inferior frontal gyrus (IFG) during novel metaphor processing, individuals with SZ showed an over-activation of the left IFG and the middle frontal gyrus (MFG). Thus, people with schizophrenia demonstrated a reversed lateralization during the processing of non-salient novel metaphorical interpretations. Only a few studies have examined processing of irony via neuroimaging in SZ patients. Utilizing fMRI, Rapp et al. (2010) investigated irony comprehension and lateralization in 15 non-clinical females with schizotypal personality traits. The results demonstrated a significant negative association between schizotypal personality traits and language lateralization in the middle temporal gyrus of both hemispheres. The authors conclude that schizotypal personality traits are associated with brain activation in language comprehension brain regions rather than ToM network.

Recently, irony comprehension in female SZ patients was tested in a fMRI study and an offline irony task (Rapp et al., 2013). Although the SZ participants made few mistakes on the offline irony test, their performance differed substantially from that of TD individuals. When processing ironic sentences SZ patients, in contrast to TD adults, have shown diminished activation in the RH temporal and parietal regions along with a decreased BOLD response in the posterior medial prefrontal cortex and the LH insula. Enhanced bilateral BOLD response was observed in the posterior temporal lobe bilaterally. Thus, evidence suggests altered brain lateralization in SZ during comprehension tasks involving figurative language, including irony, along with reduced activation in the RH.

Given the centrality of pragmatic abilities to our social participation and well-being and the compromised pragmatic abilities in SZ it is important to expand the knowledge regarding the pragmatic abilities of individuals with SZ by examining their ironic comprehension. Furthermore, atypical brain lateralization has been documented in SZ in processing sub-pragmatic ability, and in particular in processing figurative language. The current study aims to investigate whether deficient irony comprehension is associated with atypical brain lateralization in SZ. The two aims of the current study are thus (1) to investigate the comprehension of irony in adults with SZ in comparison to – age-matched TD adults using an irony questionnaire and a DVF task; and (2) to test hemispheric differences in accessing the literal versus the ironic interpretations of texts. We hypothesized that TD participants would outperform the clinical group. In accordance with previous studies (Kircher et al., 2007; Mashal et al., 2008), we predicted that we would find a RH advantage in processing ironic interpretations in TD adults, and a reversed lateralization in the SZ group.

## Materials and Methods

### Participants

Thirty-four native Hebrew speakers participated in the study, 16 adults with SZ (4 women) and 18 TD adults (9 women). There was no significant difference in age and gender distribution across groups (**Table 1**). All participants were right-handed (according to their self-report), had intact or corrected vision, reported no neurological problems, and had completed at least 11 years of school. The clinical schizophrenia diagnosis was given by a multidisciplinary hospital team using the DSM-5 (American Psychiatric Association, 2013) criteria. The participants with SZ who met these were inpatients and outpatients who had been recruited from the Geha Mental Health Center in Israel. Their clinical symptoms were evaluated with the Positive and Negative Syndrome Scale (PANSS) (Kay et al., 1987; **Table 1**). All the participants took the vocabulary subtest of the Wechsler Adult Intelligence Scale (Wechsler, 2001). In this test, participants are asked to define a given word.

**Table 1:** Demographic characteristics, by group.

Screening tests	SZ (*N* = 16)	TD (*N* = 18)	*T*	*p*
Age	31.38 (8.81)	29.50 (3.88)	0.79	0.40
Gender (male: female)	12:4	9:9	2.24^!^	0.13
Vocabulary	39.69	48.55	5.01	0.0001
PANSS (total)	70.20 (20.30)			
PANSS (positive)	13.40 (4.35)			
PANSS (negative)	19.50 (6.60)			
PANSS (general)	34.75 (10.10)			

*! = Chi-square (df = 1).*

All the participants signed an informed consent form prior to their participation in the study. The research was carried out in accordance with the Code of Ethics of the World Medical Association (Declaration of Helsinki) for experiments involving humans, as approved by the Institutional Review Boards of the Geha Mental Health Center, and was also approved by the school of education’s ethical board at Bar Ilan University.

### Experimental Tasks

The participants completed a DVF task and filled out an irony questionnaire.

### DVF Experiment

In the DVF task participants were instructed to read the passage silently, and then indicate as accurately and as quickly as possible whether the passage that ended with the target word was meaningful or not. The aim of this task was to compare irony comprehension and hemispheric processing of ironic and literal interpretations in SZ with that of TD adults.

### Stimuli

The stimulus pool consisted of 84 short passages. The final (target) word that completed the passage gave it an ironic, literal or meaningless interpretation (ironic ending *n* = 28, literal ending *n* = 28, and meaningless ending *n* = 28). The passages and the target words of all three types were matched for length. Word frequency (based on Linzen, 2009) was matched across all word types. For example, one passage read: “Exhausted after a long day at work, David planned to go to bed early. Just as he was ready for bed, he heard a knock on the front door. David opened the door and saw that some friends had come by for a visit. David said: ‘The timing is….”’ The ironic target ending was “perfect.” Another passage read: “The final exam lasted for about 3 h and included a lot of material, not all of which had been taught in class. At the end of the test the students told the teacher: ‘The test was….”’ The literal target ending was “difficult.” Another example read: “In anticipation of her husband’s return from abroad Dana had prepared a gourmet meal, so she was very disappointed when he called to say that the flight had been ….,” the meaningless target ending was “barefoot.”

### Stimulus Construction

The ironic and the literal target words were selected by 20 judges (aged 18–35), who did not participate in the experimental tasks. The judges were presented with the passages with the final word missing and were asked to write down a single word that could end each of the 84 passages either literally or ironically. Only words that were used by at least 80% of the judges were chosen for the study. Meaningless target words were created by the experimenters. Next, in order to validate the type of the passages, all the passages (including the selected target words) were presented to 20 additional judges (age 18–35). The judges were asked to indicate whether interpretation of the passage was ironic, literal, or meaningless. The agreement rates of the judges were 97% (*SD* = 0.06), 96% (*SD* = 0.81), and 92% (*SD* = 0.11), for the literal, the ironic and the meaningless endings, respectively (for further details see Saban-Bezalel and Mashal, 2015a).

### Hemispheric Procedure

The DVF experiment included 28 ironic passages, 28 literal passages, and 28 meaningless passages. The participants sat in front of a computer screen with their heads stabilized on a chin-rest at a viewing distance of 60 cm, and placed two right-hand fingers between the key that denoted that the passage was meaningful and the key that denoted that the passage was meaningless. A fixation point appeared at the center of the screen for 2000 ms, and once it disappeared the passage appeared at the center of the screen for 2500–7000 ms, depending on the number of words in the passage (presentation time was determined in the pilot study). Next, a fixation point was presented for 300 ms, after which the target word appeared and remained on the screen for 180 ms. The target words were presented 2.8 degrees to the right or to the left of the fixation point, so that processing took place in either the right visual field∖left hemisphere (RVF∖LH) or the left visual field∖right hemisphere (LVF∖RH). The fixation point remained on the screen until the target word disappeared. The participants were instructed to read the passage silently, focus on the fixation point without moving their eyes, and then indicate as accurately and as quickly as possible whether the passage was meaningful or not by pressing the designated key within 2000 ms. The session began with a practice list consisting of nine trials that were not used in the experiment. The passages were presented in random order, with a short break offered after completion of half of the experimental trials.

### Irony Questionnaire

The irony questionnaire included 15 short passages, including 10 ironic ones and 5 literal ones, presented in mixed order. The participants were asked to read each passage and write down an answer to a comprehension question. For example, “Tom and Daphne took a ride in a very crowded and stuffy bus, so that they had to stand during the ride. When they got off the bus Tom said, ‘Riding in public transportation is fun.’ What did Tom think about public transportation?” This questionnaire, which had no time limit, tests irony comprehension. The items in the Irony questionnaire were also used in the computerized test.

All the tasks were performed on the same session. The two irony tasks (the DVF experiment and the Irony questionnaire) were not performed consecutively but rather they were separated by the vocabulary task.

## Results

Reaction times for correct responses and the percentage of correct responses were calculated, after omitting the outliers (responses ± 2 SD from the mean), for each participant in all experimental conditions of interest. Only correct trials were analyzed. 8.7% of participants’ responses were omitted (no response was recorded). Responses for the meaningless endings that served as fillers were not analyzed.

Two 2X2X2 repeated-measures analyses of variance (ANOVA) were conducted, with visual field (left, right) and type of target word (literal, ironic) as within-subject factors, and group (SZ, TD) as the between-subject factor. One analysis was conducted for accuracy and another for reaction times. As the two groups differed in their vocabulary abilities, 2X2X2 repeated-measures analyses of covariance (ANCOVA) were also conducted, with visual field (left, right) and type of target word (ironic, literal) as within-subject factors, group (SZ, TD) as the between-subject factor and vocabulary scores as covariate variables. The correlations between the PANSS scores and the computerized task indices (i.e., accuracy and reaction times in each visual field separately) were also calculated. To test performance differences between the offline questionnaire and the computerized test in the SZ group, a paired-samples *t*-test was used.

### Accuracy Analysis

**FIGURE 1:**
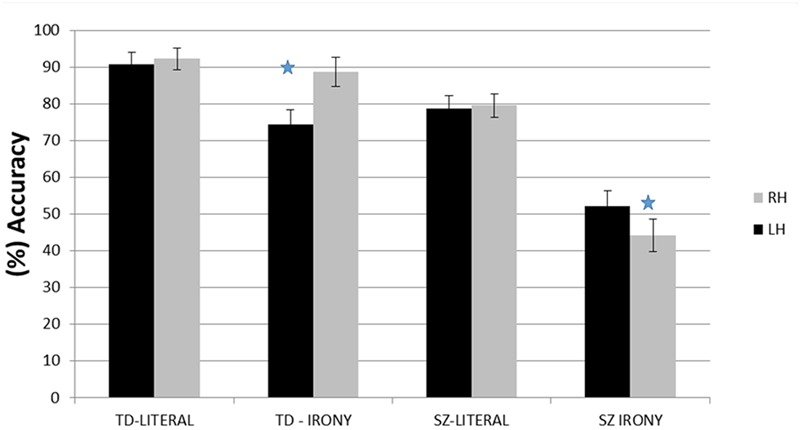
Percent of correct responses (and SE) within each hemisphere, by group and stimuli type. ^∗^*p* < 0.05.

Means and standard deviations of correct responses are presented in **Figure 1**. A significant main effect of group was found, *F*(1,34) = 34.05, *p* < 0.001, η^2^ = 0.50, with the SZ group being less accurate (*M* = 63.51%, *SD* = 2.93) than the TD group (*M* = 86.50%, *SD* = 2.62). The main effect of target word was also significant, *F*(1,34) = 48.24, *p* < 0.001, η^2^ = 0.58, indicating that responses to literal interpretations (*M* = 85.25%, *SD* = 2.17) were more accurate than those to ironic interpretations (*M* = 64.75%, *SD* = 2.72). The main effect of visual field was non-significant, *F*(1,34) = 2.82, *p* = 0.10, η^2^ = 0.07.

The two-way interaction of visual field X group was significant, *F*(1,34) = 19.09, *p* < 0.001, η^2^ = 0.36. A Bonferroni *post hoc* analysis revealed that while responses in the TD group were more accurate in the left visual field (LVF/RH) than the right visual field (RVF/LH), *p* < 0.0001, no differences between the two visual fields were found in the SZ group. The two-way interaction of target words X group was also significant, *F*(1,34) = 12.67, *p* < 0.01, η^2^ = 0.27. Bonferroni *post hoc* test revealed that in both groups responses to literal interpretations were more accurate than those to ironic interpretations, *p* < 0.05 (TD group), *p* < 0.0001 (SZ group). The two-way interaction of visual field X target word was not significant, *F*(1,34) = 0.55, *p* = 0.46, η^2^ = 0.01.

Importantly, the three-way interaction of visual field X target word X group was significant, *F*(1,34) = 14.90, *p* < 0.001, η^2^ = 0.30. A Bonferroni *post hoc* analysis revealed that, within the SZ group, responses in the RVF/LH were significantly more accurate than in the LVF/RH in processing ironic interpretations. Within the TD group, in contrast, responses to ironic endings in the LVF/RH were significantly more accurate than in the RVF/LH (**Figure 1**).

### Accuracy Analyses of Covariance

Analysis of covariance revealed that the only main effect that remained significant was that of group, *F*(1,33) = 18.32, *p* < 0.001, η^2^ = 0.36. The two-way interaction of visual field X group remained significant, *F*(1,33) = 11.29, *p* < 0.001, η^2^ = 0.33, unlike the two-way interaction of visual field X target word, which was no longer significant, *F*(1,33) = 0.02, *p* = 0.88, η^2^ = 0.00.

The three-way interaction of visual field X target word X group remained significant, *F*(1,33) = 8.56, *p* < 0.01, η^2^ = 0.20. Thus, within the SZ group, ironic interpretations were processed significantly more accurate in the RVF/LH than in the LVF/RH. In contrast, within the TD group, ironic interpretations were processed significantly more accurate in the LVF/RH than in the RVF/LH.

### RT Analysis

An analysis of reaction times revealed a significant main effect of group, *F*(1,34) = 12.17, *p* < 0.001, η^2^ = 0.26. As expected, the SZ group was slower (*M* = 1115.83, *SD* = 46.59) than the TD group (*M* = 897.69, *SD* = 41.67). The main effect of target word was also significant, *F*(1,34) = 9.53, *p* < 0.01, η^2^ = 0.22. The response times to ironic interpretations (*M* = 1045.89, *SD* = 34.59) were slower than to literal interpretations (*M* = 967.64, *SD* = 32.84). The main effect of visual field was not significant, *F*(1,34) = 4.00, *p* = 0.053, η^2^ = 0.10.

The two-way interaction of visual field X group *F*(1,34) = 4.43, *p* < 0.05, η^2^ = 0.11, was significant. Bonferroni *post hoc* analyses revealed that, within the TD group, reaction times in the LVF/RH (*M* = 853.55, *SD* = 41.14) were faster than in the RVF/LH (*M* = 941.84, *SD* = 46.81, *p* < 0.01. The two-way interaction of target word X group, *F*(1,34) = 4.53, *p* < 0.05, η^2^ = 0.11, was significant as well. A Bonferroni analysis revealed that, within the SZ group, response times to ironic interpretations (*M* = 1181.95, *SD* = 51.56) were slower than to literal interpretations (*M* = 1049.71, *SD* = 48.95), *p* < 0.01. The two-way interaction of visual field X target word was also significant, *F*(1,34) = 4.72, *p* < 0.05, η^2^ = 0.12. A Bonferroni analysis revealed faster reaction times for ironic interpretations presented in the LVF/RH (*M* = 1003.13, *SD* = 33.13) than in the RVF/LH (*M* = 1088.64, *SD* = 43.10), *p* < 0.05.

**FIGURE 2:**
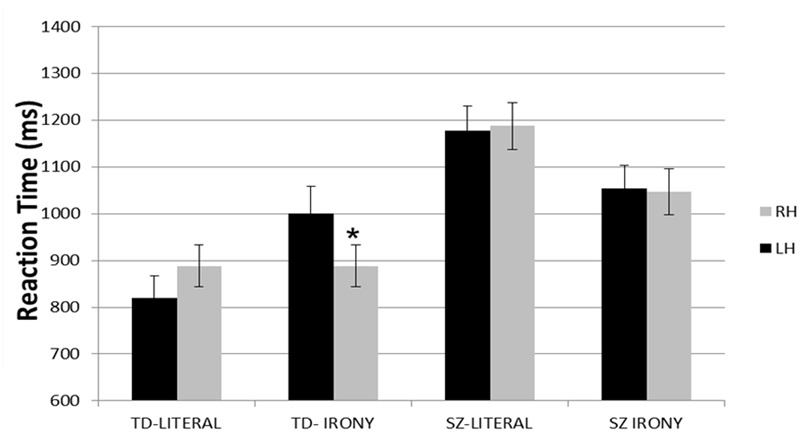
Reaction times (and SE) within each hemisphere, by group and stimulus type. ^∗^*p* < 0.05.

Importantly, the three-way interaction of visual field X target word X group was significant, *F*(1,34) = 6.90, *p* < 0.05, η^2^ = 0.17. A Bonferroni *post hoc* analysis revealed that, within the TD group, response times for ironic target words were faster in the LVF/RH than in the RVF/LH, *p* < 0.001. There were no differences between the visual fields within the TD group for the literal interpretations (*p* = 0.86) or for either ironic (*p* = 0.82) or literal (*p* = 0.85) target words in the SZ group (**Figure 2**).

### Reaction Time Analyses of Covariance

ANCOVA revealed that all the main effects were no longer significant. The two-way interaction of target word X group was significant, *F*(1,33) = 8.34, *p* < 0.01, η^2^ = 0.20, indicating that reaction times for irony were slower than for literal target words in the SZ group. In contrast, no differences were found in response times between the two types of target word in the TD group, *p* = 0.77. The two-way interactions of visual field X target word and visual field X group were no longer not significant, *F*(1,33) = 0.01, *p* = 0.9, *F*(1,33) = 1.44, *p* = 0.24, respectively.

The three-way interaction of visual field X target word X group was no longer significant, *F*(1,33) = 2.98, *p* = 0.09, η^2^ = 0.08.

The correlations between accuracy and reaction times were all negative (*rs* ranging from -0.47 to -0.76, *p* < 0.01, *p* < 0.001, respectively), indicating that there was no speed-accuracy tradeoff.

### Correlation between the PANSS and the Comprehension of Ironic and Literal Interpretations

We calculated the correlations of the PANSS scores (positive, negative, total) and the computerized task indices (accuracy and reaction times) in each visual field separately. A negative correlation was found between accuracy for ironic target words presented in the RVF/LH and the PANSS negative scores, *r* = -0.64, *p* < 0.05. Thus, as the more negative symptoms are present, the lower the comprehension of irony. All the other correlations were non-significant.

### Irony Questionnaire Analysis

We further tested whether there were performance differences between the questionnaire and the computerized test by conducting paired samples *t*-test. The scores for ironic and literal interpretations in the computerized task were calculated. The score for irony was the averaged accuracy rate of the right and the left visual field for the ironic target words. Similarly, the score for literal interpretations was the averaged accuracy rate of the right and the left visual field for the literal target words. As performance on the questionnaire was at ceiling in the TD group, the results were analyzed only for the SZ group. Paired sample *t*-tests revealed better performance on the questionnaire than on the computerized test for both the ironic items, *t*(12) = 2.83, *p* < 0.05, and the literal items, *t*(12) = 3.29, *p* < 0.01 (**Table 2**).

**Table 2:** Means and SD of correct responses on the questionnaire and the computerized task in the SZ group.

Task	Irony		Literal	
	*M*	*SD*	*M*	*SD*
Questionnaire	77.70	24.10	96.90	7.50
Computerized assessment	48.00	5.6	79.05	0.6

## Discussion

The current study investigated irony comprehension and hemispheric processing for irony in SZ as compared to healthy adults. The study reveals three main findings: first, a comparison between the two tasks that tested irony comprehension indicates that SZ have a certain ability to comprehend irony but this capacity depends on the task characteristics. Second, SZ demonstrated an atypical reversed hemispheric lateralization when processing ironic interpretations as compared to healthy adults. Whereas TD participates showed the expected RH advantage in processing ironic passages, responses to ironic target words presented to the RVF/LH were more accurate than to the LVF/RH for the SZ group. Lower comprehension of irony in the LH also correlated with greater negative symptoms.

In line with previous studies showing deficits in irony comprehension in SZ (e.g., Colle et al., 2013; Schnell et al., 2016) the performance of the SZ patients in the current study was poorer than that of healthy adults. Nonetheless, our findings indicate that SZ show good ability to comprehend irony when there are no time constraints. Their accuracy rates were higher for the irony questionnaire (*M* = 77.70%) than for the computerized task (*M* = 48%). These differences were also found for literal stimuli, where their accuracy was high (*M* = 97%) on both the offline questionnaire and the computerized task (*M* = 79%). Thus, participants with schizophrenia exhibit better performance for irony interpretations when there are no time constraints. Our findings corroborates with previous studies that have shown that when they are given a linguistic clue (Schnell et al., 2016) or under certain conditions (Ziv et al., 2011) SZ can comprehend irony. Thus, in addition to cognitive and ToM ability, which have been found to be associated with irony comprehension in SZ (Champagne-Lavau and Stip, 2010; Varga et al., 2014), the type of task might also influence performance as well. We acknowledge that irony processing in everyday situations are performed in real time with time restrictions, so that the capabilities the participants demonstrated in this study will not necessarily be reflected in their everyday life.

Healthy participants exhibited the expected hemispheric asymmetry in processing ironic interpretations. Their accuracy rate was higher for ironic interpretations presented in the LVF/RH than in the RVF/LH, while their reaction times in the LVF/RH were faster than in the RVF/LH. Since the comprehending irony may require activating broader semantic fields, including non-salient meanings, the findings support both the Fine vs. Coarse Semantic Coding Theory (Beeman, 1998) and the GSH (Giora, 1997, 2003). Thus, in accordance with the FCSCT, after encountering the ironic target word the RH engaged in coarse semantic coding, and weakly activated large semantic fields containing multiple alternative meanings. Since the ironic interpretation is usually more semantically distant than the literal meaning, RH semantic processes are indeed better suited for irony comprehension. In contrast, no hemispheric differences were found for literal interpretations. The literal target words might have been accessible to both hemispheres due to the simplicity of the task.

Unlike our healthy participants, the SZ demonstrated atypical reversed lateralization to ironic stimuli. That is, the LH was significantly more accurate for ironic stimuli than the RH. These hemispheric differences cannot be attributed to differences in vocabulary scores, as the latter did not affect the results, meaning that vocabulary abilities as tested in the current study cannot explain the differences between the groups. These findings are in accordance with previous findings of studies that investigated figurative language comprehension in patients with schizophrenia (Kircher et al., 2007; Mashal et al., 2013; Rapp et al., 2013) and reinforce the suggestion that SZ possess altered hemispheric processing as compared to healthy adults. Mashal et al. (2013) attributed their findings of the inefficient processing of novel metaphors in SZ to compensation processes. Our findings are in line with the Right Hemisphere Dysfunction theory (Mitchell and Crow, 2005). This theory posits that due to a decrease in LH function, the brain reorganizes itself and homologous structures in the RH mediate the damaged function. This reorganization causes the standard functions of the RH to be compromised. Hence, the somewhat better performance in the left-hemisphere processing of irony may be due to compensation or the recruitment of additional cognitive resources (e.g., working memory, attributed to the LH) to increase their comprehension of irony (Mashal et al., 2013). Atypical hemispheric processing for irony was also observed in a recent study (Rapp et al., 2013). The results revealed lower activation in right temporal brain regions as well as in the posterior medial prefrontal cortex in a group of females with SZ for ironic stimuli. The activation of the medial prefrontal cortex has been ascribed to conflict monitoring and making judgements about the external world. Taken together, the paucity of studies that tested irony comprehension in SZ suggest RH dysfunction in SZ during irony comprehension together with difficulties in simulating a social situation during the decision process to judge whether a passage make sense or not.

The LH’s ability to comprehend irony in SZ patients correlated negatively with the patients’ negative symptoms. That is, irony comprehension decreased as negative symptoms increased. Negative symptoms are considered to be an outcome of insufficient capacity for representing intentions (Corcoran, 2001; Salvatore et al., 2008). Our finding matches previous findings that have found a link between negative symptoms and ToM (Corcoran, 2001; Urbach et al., 2013) and between SZ symptoms and the ability to recognize and repair communicative failures (Bosco et al., 2012b). Irony comprehension relies on perception and comprehension of the conflict between the context (what has been said) and the speaker‘s intentions. Indeed, it has been suggested that the deficient comprehension of irony and sarcasm in SZ is due to deficits in ToM and perspective-taking (Kosmidis et al., 2008; Champagne-Lavau and Stip, 2010). Thus, our findings support previous studies showing a connection between SZ symptoms and the comprehension of figurative language (Mashal et al., 2013).

We need to mention some of the study’s limitations and suggest future research in the present field. The schizophrenia group was composed of in- and outpatients. Due to the small size of group we did not test the difference between the two subgroups (in- and outpatients). The fact that some of the participants were inpatients may indicate temporary imbalances that could have affected their performance. In light of the importance of this field to social understanding, we believe that further studies are needed to explore irony comprehension in SZ. We encourage the development of intervention programs in the field of social cognition in general and irony comprehension in particular, with the aim of enhancing SZ’s quality of life (Saban-Bezalel and Mashal, 2015b). For instance, a recent pilot study demonstrated the efficacy of a 20 sessions Cognitive-Pragmatic Treatment (CPT) program that focuses on improving different aspects of communication (e.g., facial expression recognition, social appropriateness and conversational rules) in SZ (Bosco et al., 2016). The program improved patients’ performance in several communication domains including, linguistic, extralinguistic, paralinguistic and social appropriateness. Furthermore, following the CPT, the behavioral changes were associated with increased activation in the superior, inferior, and medial frontal gyri, as well as in the superior temporal gyri as was documented in a single case patient (Gabbatore et al., 2017). Thus, it seems that individuals with SZ are able to improve their cognitive and social cognitive abilities following cognitive training. However, further research on larger samples is necessary to establish the behavioral changes and to confirm the neural modifications.

To summarize, the present study emphasizes the atypical hemispheric processing in patients with schizophrenia as a possible reason for their deficient irony processing. Improved irony comprehension is associated with decreased negative symptoms. In addition, despite SZ patients’ known difficulty in comprehending irony, we found that their performance is fairly good under conditions that do not involve time restrictions.

## Author Contributions

RS-B stimulus construction, data collection, data analysis, writing article. NM project idea, study design, data analysis, manuscript revision.

## Conflict of Interest Statement

The authors declare that the research was conducted in the absence of any commercial or financial relationships that could be construed as a potential conflict of interest.
